# Effectiveness of combination therapy with ISA101 vaccine for the treatment of human papillomavirus-induced cervical cancer

**DOI:** 10.3389/fonc.2022.990877

**Published:** 2022-10-10

**Authors:** Haigang Ding, Juan Zhang, Feng Zhang, Yan Xu, Yijun Yu, Wenqing Liang, Qingping Li

**Affiliations:** ^1^ Department of Gynecology, Shaoxing Maternity and Child Health Care Hospital, Shaoxing, China; ^2^ Obstetrics and Gynecology Hospital of Shaoxing University, Shaoxing, China; ^3^ Intensive Care Unit, Zhoushan Hospital of Traditional Chinese Medicine Affiliated to Zhejiang Chinese Medical University, Zhoushan, China; ^4^ Medical Research Center, Zhoushan Hospital of Traditional Chinese Medicine Affiliated to Zhejiang Chinese Medical University, Zhoushan, China

**Keywords:** cervical cancer, vaccine, ISA101, immunotherapy, human papilloma virus

## Abstract

Cervical cancer is one of the women-associated tumors that affects numerous people yearly. It is the fourth most common malignancy in women worldwide. Following early diagnosis, this cancer can be cured mainly by traditional methods such as surgery, tumor resection, and chemotherapy; nonetheless, it becomes more challenging to treat in advanced and metastatic stages. With the advent of novel treatments such as angiogenesis inhibitors or immuno-checkpoint blockers in recent years, the survival rate of patients with advanced cervical cancer has significantly increased. However, it has not yet reached a satisfactory level. It has been revealed that human papillomavirus (HPV) infection is responsible for more than 90% of cervical cancer cases. However, evidence revealed that monotherapy with anti-HPV vaccines such as ISA101 could not affect tumor growth and progression in patients with HPV-induced cervical cancer. Therefore, combining ISA101 and immune checkpoint blockers or other immunotherapeutic approaches may be more robust and effective than monotherapy with ISA101 or immune checkpoint blockers for treating cervical cancer. This review summarizes the ISA101 properties, advantages and disadvantages. Furthermore, various conducted combination therapies with ISA101 and the effectiveness and challenges of this treatment have been discussed.

## Introduction

Cervical cancer is a principal public health challenge for women (1). After breast, colorectal, and lung malignancies, cervical cancer is the fourth reported cancer incidence. It accounts for around 7.5% of all women’s cancer death worldwide (2–4). Usually, the age of cervical cancer diagnosis in women is 53, the age of death of these people is 59 years, and the survival rate is about 5.5 years (3, 5). Federation of Gynecology and Obstetrics (FIGO) 2018 staging system for cervical cancer is shown in **Table 1** (6).

**Table 1:** FIGO staging of cervical cancer (2018).

Stage	Description
**I**	• The carcinoma is firmly limited to the cervix (extension to the uterine corpus must be ignored)
**IA**	• Invasive carcinoma is only diagnosed by microscopic examination • Maximum depth of invasion lower than 5 mm
**IA1**	• Measured stromal invasion lower than 3 mm in depth
**IA2**	• Measured stromal invasion greater than or equal to 3 mm and lower than 5 mm in depth
**IB**	• Invasive carcinoma with measured deepest invasion greater than or equal to 5 mm (greater than Stage IA) • Lesion are limited to the cervix uteri
**IB1**	• Invasive carcinoma greater than or equal to 5 mm depth of stromal invasion, and lower than 2 cm in greatest dimension
**IB2**	• Invasive carcinoma greater than or equal to 2 cm and lower than 4 cm in greatest dimension
**IB3**	• Invasive carcinoma greater than or equal to 4 cm in greatest dimension
**II**	• The carcinoma invades beyond the uterus • The carcinoma has not extended onto the lower third of the vagina or to the pelvic wall
**IIA**	• Tumor limited to the upper two-thirds of the vagina • Parametrial is not involved in this stage
**IIA1**	• Invasive carcinoma lower than 4 cm in greatest dimension
**IIA2**	• Invasive carcinoma greater than or equal to 4 cm in greatest dimension
**IIB**	• Parametrial is affected but not up to the pelvic wall
**III**	• The carcinoma involves the lower third of the vagina • Extends to the pelvic wall leading to hydronephrosis or nonfunctioning kidney • Involvement of pelvic and/or para-aortic lymph nodes
**IIIA**	• The carcinoma involves the lower third of the vagina • The pelvic wall is not affected
**IIIB**	• Extension to the pelvic wall • Hydronephrosis or nonfunctioning kidney
**IIIC**	• The pelvic and/or para-aortic lymph nodes are involved • Regardless of tumor size and extent
**IIIC1**	• Pelvic lymph node metastasis only
**IIIC2**	• Para-aortic lymph node metastasis
**IV**	• The carcinoma has extended beyond the true pelvis or has involved (biopsy proven) the mucosa of the bladder or rectum * A bullous edema, as such, does not permit a case to be allotted to this stage
**IVA**	• Tumor spreads to adjacent pelvic organs

Evidence demonstrated that HPV strain type and epigenetics are the main pathogenic risk factors accountable for advanced cervical cancers. In HPV, E6 and E7 proteins lead to the proliferation and growth of cervical tumor cells (7). Notwithstanding the increasing efforts to improve therapy in cervical cancer, the majority of women still die, generally from the chemoresistant or recurrent disease (8). Surgery, radiotherapy, chemotherapy, targeted drug therapy, and immunotherapy are the available therapeutic approaches to treat cervical cancer. However, monotherapy with these methods has not been successful. Therefore, researchers are trying to design and employ novel combination therapies, such as combining cancer vaccines with immune checkpoint blockers (9, 10).

ISA101 is one of the most effective vaccines containing synthetic peptides E6 and E7 HPV-16. After the vaccine is injected, the peptides are captured and processed by dendritic cells (DCs). In the next step, these processed antigens are presented to specific CD4^+^ and CD8^+^ T cells, ultimately triggering anti-tumor responses (11). According to available studies, the ISA101 vaccine may be involved in the clearance of peritumoral lesions. In this regard, this vaccine can eradicate HPV-16 ^+^ pre-malignant vulvar lesions, which are associated with the strength of T cell-dependent responses. Although this vaccine can elicit immune responses, monotherapy clinical outcomes show its ineffectiveness due to barriers such as immunosuppressive tumor microenvironment (TME) and inhibitory components of the immune system (12). Therefore, combining ISA101 with other agents used in immunotherapy, such as immune checkpoint blockers, can increase the effectiveness of treatment. This review discusses the ISA101 vaccine properties, its effect on treating cervical cancer, and the chances of increasing its effectiveness by employing various combination therapies.

## HPV and cervical cancer

**Figure 1:**
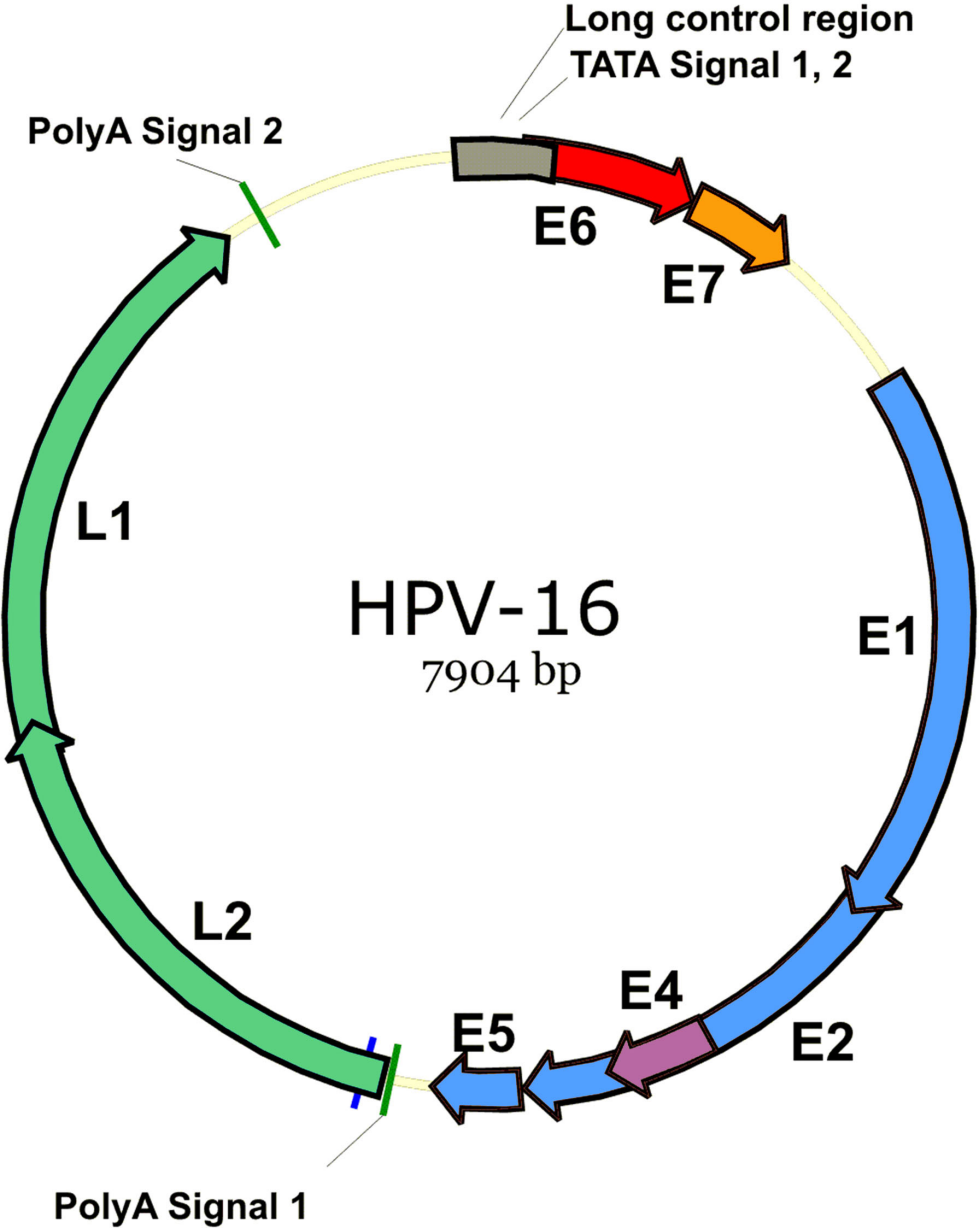
HPV-16 genome. HPV-16 is a relatively small, uncoated virus containing the L1 and L2 capsid proteins and the E proteins involved in proliferation and tumorigenesis (E1, E2, E4, E5, E6, E7), of which E6 and E7 are associated with tumorigenesis in cervical cancer.

**Figure 2:**
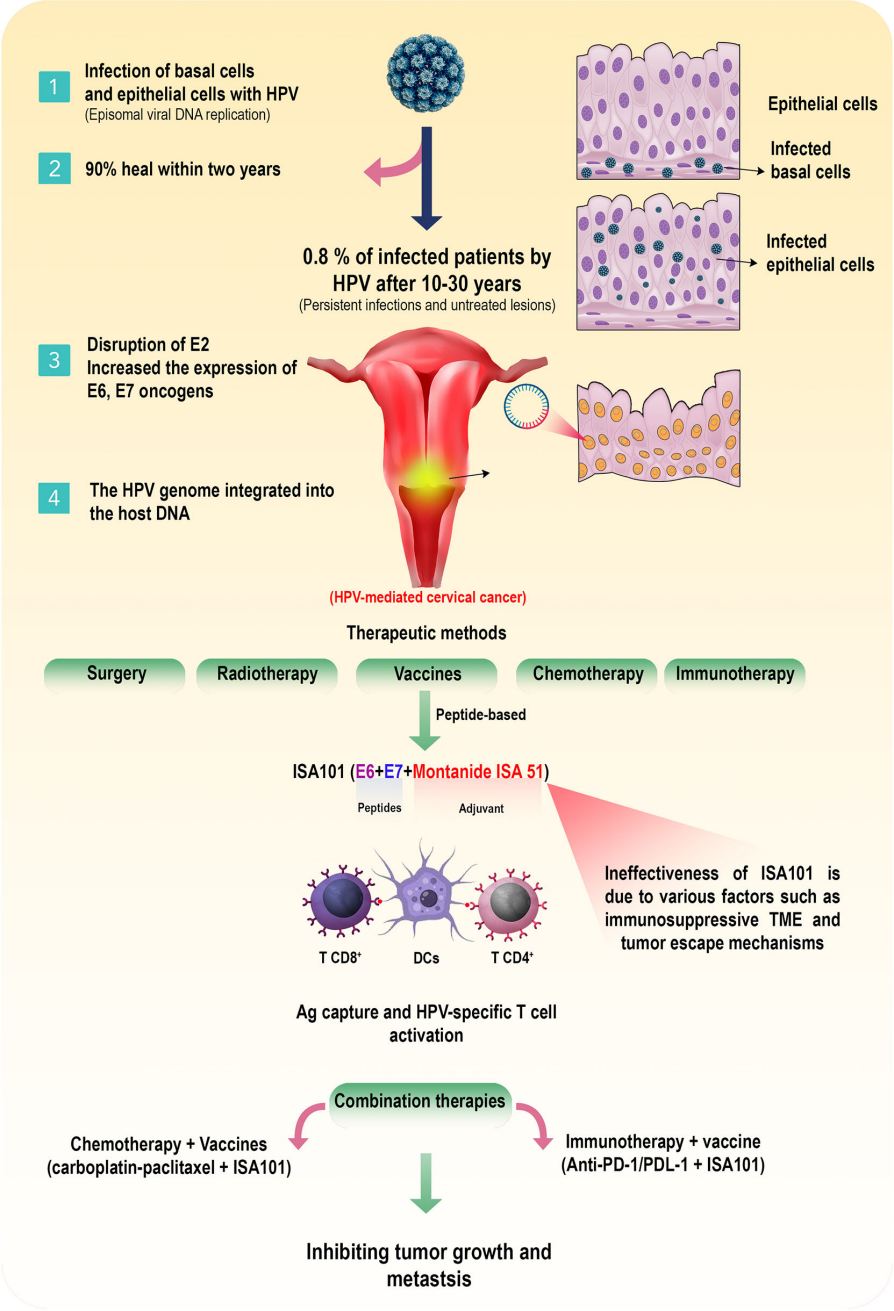
HPV-induced cervical cancer and available therapeutic approaches. Following infection with HPV-16 and HPV-18 subtypes, the basal cells in the cervix are affected, and after a few weeks, the infection spreads to the epithelial cells and HPV replicate in the cytoplasm of these cells (episomal viral DNA replication). Regularly, most people recover spontaneously after a period of one to two years. However, after 10 to 30 years, in about 0.08% of patients, persistent infections along with untreated lesions could be associated with disruption of E2 and E6/E7 oncogenes upregulation and the HPV genome integration into the host DNA, resulting in HPV-induced cervical cancer. There are various treatments such as surgery, radiotherapy, chemotherapy, and vaccination for these patients. Among the peptide-based vaccines, the ISA101 vaccine, which consists of two synthetic proteins, E6 and E7, along with Montanide ISA 51 adjuvant, can activate the specific responses of T cells against HPV-infected cells. Although due to obstacles such as the immunosuppressive TME and other unknown reasons, this vaccine is not effective. Therefore, combining of chemotherapy and other immunotherapeutic approaches, such as immune checkpoint blockers with ISA101 could be more effective than ISA101 monotherapy for advanced cervical cancer.

In recent decades, studies on cervical cancer have revealed that the disease can be associated with infection with specific HPV serotypes (13). HPV is a member of the *Papovaviridae* family and a relatively small, uncoated virus containing the L1 and L2 capsid proteins and the E proteins involved in proliferation and tumorigenesis (E1, E2, E4, E5, E6, E7) of which E6 and E7 are associated with tumorigenesis in cervical cancer (7, 14) (**Figure 1**). So far, more than 200 types of HPV have been identified, about 30 of which are sexually transmitted and can mainly infect the cervix, vulva, vagina, anus, and penis (15). Fifteen HPV subtypes are associated with cervical cancer, and HPV types 16 and 18 are responsible for about 70% of the disease. Although the majority of HPV16 infections are self-limited and not associated with any symptoms in some infected patients, the HPV16 genome can be integrated into the host cells’ DNA, resulting in persistent infections and pre-neoplastic lesions formation (16, 17) (**Figure 2**). On the other hand, cervical adenocarcinomas are often age-related and less commonly associated with HPV infection in people over 60 years of age (18). However, current investigations disclosed that after HPV16, HPV18 could consider the second most oncogenic HPV genotype (approximately 10% of cases). HPV18 is highly involved in adenocarcinoma and adeno/adenosquamous *in situ* (19). Furthermore, HPV18-induced cervical cancer has the worst prognosis among other types of HPV (20, 21).

Based on the association with cervical cancer and its precursor lesions, genital HPV types are categorized into non-oncogenic HPV types (6, 11, 42, 43 and 44) ​​and oncogenic types (16, 18, 31, 33, 35, 39, 45, 51, 52, 56, 58, 59, 68, 73 and 82) (15). However, non-oncogenic subtypes are also sometimes detected in cervical cancer. HPV usually affects the cutaneous mucosal epithelium, and viral particles begin to replicate in mature epithelial cells. Disruption of the normal cell cycle and stimulation of uncontrolled cell division by the virus causes genetic damage (22). In most healthy people, cervical changes following an HPV infection are transient and resolve spontaneously within one to three years.

Nonetheless, factors such as genetics, polymorphisms in genes involved in infection clearance, coinfections, recurrent mutations and the development of new HPV variants increase the risk of cervical cancer. In addition, individual hormonal patterns, impaired antiviral immune responses, and recurrent infections are other risk factors for cervical cancer (13). Therefore, diagnosing and preventing HPV infection is vital in reducing the risk of cervical cancer. Although this type of infection does not always lead to cancer, various factors are involved in this process, many of which are unknown.

## Treatment of cervical cancer

Based on available knowledge, the selection of cancer therapy methods in patients with cervical carcinoma *in situ* or stages I–IV cervical cancer is different. Primary cancer therapies are surgery, radiotherapy, and chemotherapy (1, 23). Surgery and radiotherapy are frequently employed to treat small-scale or early carcinoma, eradicating cell infection and achieving recovery. Moreover, interventional chemotherapy with paclitaxel, cisplatin, and carboplatin as co-treatment is used for the IIA and IB stages of cervical cancer (24). However, these therapeutic methods are usually limited due to resistance to treatment, damage to normal cells adjacent to the tumor, and various side effects. Therefore, HPV vaccines could be a potential approach for treating HPV infection and HPV-mediated cervical cancer (25, 26).

### HPV vaccines

Traditionally, vaccines have been developed using pathogenic microorganisms such as bacteria and viruses that are attenuated or killed, and their antigens are used to stimulate immune responses (27). The bivalent (HPV-16,18) Cervarix and quadrivalent (HPV-6,11,16,18) Gardasil vaccines are immunogenic and safe; however, this stimulation of the immune system is more of the humoral immune response and cannot be effective in anti-tumor defense (28–30). On the other hand, using cancer-associated antigens or tumor cell homogenates may increase the risk of autoimmunity (31). Consequently, novel peptide-based vaccines that stimulate the cellular immune system are used in cancer therapy, and these vaccines are being developed and tested in clinical trials (32).

In HPV-induced cervical cancer, peptide-based vaccines constructed with non-autoimmunogenic cancer-associated antigens are attractive strategies. One of the advantages of peptide-based vaccines is the convenient production process and high storage and transportation stability. However, these vaccines have low stability and immunogenicity *in vivo*, which are the disadvantages of this type of vaccine (33). Several peptide-based vaccines are being evaluated, including HPV-16 E7_11-20/86-93_ (34), HPV-16 _E712-20/86-93_ (35), WT-1 (36), P16_37-63_ (37), PepCan (38), PDS0101 (39), DPX-E7 (40), five peptides (FOXM1-262, MELK-87-7N, HJURP-408, VEGFR-1-1084 and VEGFR-2-169 in Montanide ISA51 adjuvant) (41), and ISA101 (42). Among the mentioned peptide-based vaccines, we have studied the characteristics and effectiveness of the ISA101 vaccine and discussed the outcomes, advantages and disadvantages of combination therapies performed with this vaccine in the treatment of cervical cancer.

### Immunotherapeutic approaches

Cancer immunotherapy is a novel treatment option that has yielded satisfactory results in recent years. Since most cases of cervical cancer are caused by HPV infection, the integration of viral oncoproteins E6 and E7 in the genome of host cells and uncontrolled expression of these proteins increases the probability of genetic mutations and immune escape (15, 43). On the other hand, suppressing the expression of inhibitory molecules such as programmed cell death ligand-1 (PDL-1) by tumor microenvironment (TME) components and inducing anti-tumor immune responses are the foundation of cervical cancer immunotherapy (44, 45). Pembrolizumab (anti-PDL-1 antibody) was the first US Food and Drug Administration (FDA) approved immunotherapy in 2018 for patients with PDL-1^+^ or resistant/metastatic cervical cancer. Other novels FDA-approved immunotherapies are pembrolizumab in combination with bevacizumab and chemotherapy for first-line PDL-1^+^ persistent or resistant/metastatic cervical cancer and tisotumab vedotin for second-line (46). Other novel drugs such as balstimab, cadonilimab, camrelizumab, cemiplimab, gepatanolimab, nivolumab, prolgolimab, sintilimab, zimberelimab, atezolizumab, durvalumab, IBI-310, ipilimumab, and zafrelimab often in the category of immune checkpoint blockers, are also being administered in different phases of clinical trials on patients with cervical cancer (47). Most drugs target the PD-1/PD-L1 and cytotoxic T-lymphocyte-associated protein 4 (CTLA-4) axis. Despite the satisfactory outcomes obtained from immune checkpoint blockers in the treatment of cervical cancer, the effectiveness of monotherapy with these blockers, as well as their toxicity and safety, still need more investigations in the future.

## ISA101 vaccine

ISA101 vaccine comprises 13 long synthetic peptides with 25 to 35 amino acids representing the entire sequence of HPV-16-derived E6 and E7 oncoproteins administered intradermally or subcutaneously (**Table 2**). The SA101 comprised nine E6 and four E7 synthetic peptides that could be dissolved in dimethyl sulfoxide (DMSO) and phosphate-buffered saline (pH 7.5) and emulsified with incomplete Freund’s adjuvant (Montanide ISA 51) (48). These long synthetic peptides are effectively captured by DCs and, following antigen processing, presented to HPV-16-specific CD4^+^ and CD8^+^ T cells, inducing anti-HPV immune responses. Clinical trials demonstrated that the administration of ISA101 led to constant immune stimulation in patients with advanced cervical cancer with minimal toxicity (42). Enzyme-linked immunosorbent spot (ELISpot) analysis demonstrated that a combination of E6 and E7 peptides in ISA101 could induce a robust T cell-mediated immune response, releasing interferon-gamma (IFN-γ) (49). The induced T cell-specific responses following two doses of 50 µg vaccine can last up to about a year (50). Reported adverse effects of ISA101 administration are local swelling (100%), fever (64%), and transient influenza-like symptoms (48). Although the vaccine effectively stimulates cellular immune responses, the results show that in most patients with advanced cervical cancer, ISA101 alone cannot lead to tumor regression, and some patients pass away due to tumor growth. However, ISA101 can be effective in cervical intraepithelial neoplasia (51). Accordingly, monotherapy with ISA101 does not seem to succeed in tumor regression, and combination therapies with this vaccine can overcome its weaknesses in patients with advanced cervical cancer.

**Table 2:** The whole sequence of HPV-16-derived E6 and E7 oncoproteins in ISA101.

**E6_1-32_**: *MHQKRTAMFQDPQERPRKLPQLCTELQTTIHD* **E6_19-50_**: *LPQLCTELQTTI HDIILECVYCKQQLLRREVY* **E6_41-65_**: *KQQLLRREVYDFAFRDLCIVYRDGN* **E6_55-80_**: *RDLCIVYRDGNPYAVCDKCLKFYSKI* **E6_71-95_**: *DKCLKFYSKISEYRHYCYSLYGTTL* **E6_85-109_**: *HYCYSLYGTTLEQQYNKPLCDLLIR* **E6_91-120_**: *YGTTLEQQYNKPLCDLLIRCINCQKPLCPEEK* **E6_109-140_**: *RCINCQKPLCPEEKQRHLDKKQRFHNIRGRWT* **E6_127-158_**: *DKKQRFHNIRGRWTGRCMSCCRSSRTRRETQL* **E7_1-35_**: *MHGDTPTLHEYMLDLQPETTDLYCYEQLNDSSEEE* **E7_22-56_**: *LYCYEQLNDSSEEEDEIDGPAGQAEPDRAHYNIVT* **E7_43-77_**: *GQAEPDRAHYNIVTFCCKCDSTLRLCVQSTHVDIR* **E7_64-98_**: *TLRLCVQSTHVDIRTLEDLLMGTLGIVCPICSQKP*

Several vaccine platforms for HPV-induced cervical cancer are under investigation. Here, some of the effectiveness of the most important platforms have been compared with ISA101. However, comparative studies between different platforms of anti-HPV vaccines in the normalized patients with the same combined regimen and disease stages have not been performed. Based on the available studies, DNA vaccines such as VGX-3100 and GX-188e can be even more effective than ISA101 in some cases; however, their low immunogenicity and the need for special equipment and additional adjuvants to improve their immunogenicity limit their use compared with ISA101 (10, 52, 53). RNA-based vaccines also are other options against HPV-induced cancers, which have been less studied due to the new production technology. In the animal model of cervical cancer, HPV16 RNA-LPX, in combination with a checkpoint inhibitor, showed satisfactory outcomes and led to complete regression of tumors (54). However, there is no completed clinical trial to compare the effectiveness of RNA-based vaccines with ISA101. Because of less stability, more toxicity and other limitations of RNA-based vaccines, ISA101 might be a more suitable option in HPV-induced cancers (55).

It seems that peptide-based vaccines such as ISA101 do not significantly differ in mechanism and efficacy in patients with cervical cancer or other HPV-positive cancers. Although ISA101 alone has had satisfactory results in cervical intraepithelial neoplasia, it cannot have the necessary effectiveness in patients with advanced cervical cancer (56). On the other hand, peptide-based vaccines, unlike protein-based vaccines such as Tissue antigen-cervical intraepithelial neoplasia (TA-CIN) and CIGB550-E7 fusion protein, cannot induce both cellular and humoral responses (57, 58). Moreover, according to the clinical trials, ISA101could be safer than bacterial vector-based vaccines such as ADXS11-001 because these vaccines can induce robust immune responses, which are sometimes destructive. For instance, a phase I trial (NCT01116245) of ADXS11-001 was terminated due to systemic listeriosis (59). Additionally, ISA101 may be a better option than cell-based platforms because it has a more effortless and cheaper production process and does not have the limitations of cell-based vaccines such as major histocompatibility complex (MHC) restriction. Besides, according to the previous studies, the effectiveness of ISA101 in patients with cervical cancer was higher than, for example, BVAC-C (56, 60).

However, despite the satisfactory responses of the mentioned platforms, these vaccines alone usually do not have the necessary effectiveness and combining these vaccines with other antitumor agents can significantly increase their effectiveness (61).

## Combination therapies with ISA101

As discussed, monotherapy with E6 and E7-based vaccination cannot effectively treat HPV-induced cervical cancer. To overcome this problem, combining the vaccine with other anti-tumor agents such as chemotherapy and immunotherapy can increase the effectiveness of treatment and tumor removal in a synergistic state (**Figure 2**) (**Table 3**). For instance, an investigation combining pembrolizumab and an HPV E6/E7 DNA vaccine (GX-188E) increased anti-tumor activity of the immune system with minimal toxicity in patients with advanced or recurrent cervical cancer (64). An investigation (phase II trial) demonstrated that combining ISA101 with immune checkpoint antibodies such as nivolumab (anti-PD-1) induced the anti-tumor activity of HPV-16-specific T cells and modulated immunosuppressive signals in the TME in patients with HPV-16^+^ cervical cancer. The reported adverse effects in the studied patients were elevation of transaminase and lipase, fever, injection site reaction, nausea, and fatigue (62).

**Table 3:** Combination therapies using ISA101 in HPV-induced cervical cancer.

Intervention	Type of Study	Status of the clinical trial	Sponsor of the study	Details	Research findings	Clinical outcomes	Ref(NCT number)
ISA101+ nivolumab	Clinical trial phase II	Completed	MD Anderson Cancer Center	(HPV)-16 positive solid tumors including oropharyngeal squamous cell carcinoma (OPSCC), cervical, vulvar, vaginal, anal, penile cancer ECOG performance status of </= 1	• Inducing anti-tumor activity of HPV-16-specific T cells • Modulating immunosuppressive signals in the TME in patients with HPV-16^+^ cervical cancer • The reported adverse effects were elevation of transaminase and lipase, fever, injection site reaction, nausea, and fatigue • The frequency of infiltrated CD3^+^CD8^+^PD-1^+^ cytotoxic T cells and CD68^+^PD-L1^−^ and CD68^+^PD-L1^+^ macrophages in the TME were significantly increased and directly associated with clinical response • Clinical response was correlated with the expression of IFN-γ response-associated genes	• 33% response rate in patients with HPV16^+^ cervical cancer • The reported median and three-year overall survival were 15.3 months in the studied patients	(10, 56, 62) NCT02426892
ISA101+ Carboplatin-paclitaxel	Clinical trial phase I/II	Completed	ISA Pharmaceuticals	Late-stage HPV16+ cervical cancer	• Robust vaccine-induced HPV16-specific T cell responses confirmed by high levels of interferon-γ	• A positive and significant correlation was detected between the strength of the vaccine-induced immune response and overall survival • A significantly high proportion of patients survived beyond two years after the start of the treatment	(63) NCT02128126
ISA101+ Carboplatin-paclitaxel	Clinical study	–	–	Advanced, recurrent, or metastatic cervical cancer	• Depleting immunosuppressive myeloid cells • Improving HPV-16-specific T cell	• Tumor regressions were detected in 43% of 72 patients • In 21 of the 62 studied patients, the reduction of myeloid suppressive cells caused by carboplatin and paclitaxel was linked to a low frequency of spontaneous HPV16-specific immunity. • Th1-mediated responses to the ISA101 were detected across all doses • The survival rate was prolonged in a portion of patients with higher than median vaccine-induced immune responses	(12)
ISA101+ imiquimod	A multicenter open-label, randomized controlled trial	Completed	–	HPV16-induced high-grade vulvar and vaginal intraepithelial neoplasia	• Not effective in improving CD8^+^ T-cell responses in non-responder patients • Imiquimod did not affect increasing the performance of the vaccine	• 18 of 34 patients exhibited vaccine-mediated clinical responses at three months, and 15 of 29 patients, 8 of whom exhibited a complete histologic response, at 12 months after the last vaccination • All but one of the patients who had complete histologic clearance experienced viral clearance.	(11)
ISA101B+ Cemiplimab (anti-PD-1)	Clinical trial phase II	Recruiting	Regeneron Pharmaceuticals	Patients with recurrent/metastatic HPV16 cervical cancer who have experienced disease progression after first-line chemotherapy		–	NCT04646005

ECOG, Eastern Cooperative Oncology Group.

Moreover, combining ISA101 and nivolumab showed a 33% response rate in patients with HPV-16^+^ cervical cancer. Patients received 3 mg/kg nivolumab every two weeks beginning day 8 for up to 1 year and 100 µg ISA101 on days 1, 22, and 50. The reported median and three-year overall survival were 15.3 months in the studied patients. The frequency of infiltrated CD3^+^CD8^+^PD-1^+^ cytotoxic T cells and CD68^+^PD-L1^-^ and CD68^+^PD-L1^+^ macrophages in the TME was significantly increased and was directly associated with clinical response. Furthermore, clinical response was correlated with the expression of IFN-γ response-associated genes (10). However, the mentioned adverse events led to the stoppage of nivolumab therapy (56). ISA101 is also effective in HPV16^+^ high-grade vulvar intraepithelial neoplasia *via* activation of antigen-specific T cells, regressing tumor lesions in vaccinated patients (11).

Nevertheless, in patients with HPV16-induced metastatic cervical cancer, ISA101 could not robustly stimulate antigen-specific T cell responses. Analysis of peripheral blood mononuclear cells (PBMCs) in these patients demonstrated a reduction of the stimulatory properties of APCs, resulting in low activation of T cells. In addition, the frequency of myeloid cells increased, leading to the formation of an immunosuppressive milieu. In this regard, administering carboplatin-paclitaxel in these patients led to regulating their immune profile (12).

An experimental study reported that carboplatin-paclitaxel chemotherapy in the HPV16^+^ mouse tumor model increased the frequency of myeloid cells in the circulatory and TME. Combining chemotherapy with ISA101 in patients with metastatic cervical cancer and HPV16^+^ mouse tumor model improved anti-tumor immune responses *via* depletion of myeloid cells and modulation of immunosuppressive TME (65). Another study also demonstrated that carboplatin/paclitaxel chemotherapy could deplete immunosuppressive myeloid cells, improving HPV-16-specific T cells and increasing the effectiveness of ISA101 and tumor regression in patients with HPV-16-induced cervical cancer (12). According to studies on the effectiveness of the ISA101 in patients with cervical cancer, tumor regression is realized in about 50% of vaccinated patients.

The insufficient effect of ISA101 treatment is due to barriers such as infiltration of immunosuppressive myeloid cells, regulatory T cells (Tregs), exhaustion of effector T cells, and the expression of inhibitory molecules on tumor and tumor-associated cells (10, 66). Therefore, combining chemotherapy and immunotherapy could be a practical approach *via* depletion of immunosuppressive myeloid cells, improving vaccine-induced T cell responses and overall survival in patients with HPV-16-induced cervical cancer (67). Some patients with HPV16-induced high-grade vulvar and vaginal intraepithelial neoplasia are non-responders, and CD8^+^ T cell-mediated immune responses are ineffective in viral and tumor clearance. It has been theorized that applying imiquimod at the injection site as a combination therapy could increase the vaccine’s effectiveness by improving CD8^+^ T-cell responses in non-responder individuals. However, the results showed that imiquimod did not affect increasing vaccine performance (11).

Therefore, it seems that ISA101, combined with other immunotherapeutic factors, can increase the effectiveness of treatment. However, more studies are needed in this field.

## Achievements and challenges of HPV vaccines

Given the importance of cervical cancer due to HPV infection, HPV vaccines seem necessary to reduce the burden of this type of malignancy (68–71). E6/E7-based vaccination can remarkably increase splenic and tumor IFN-γ-producing CD4^+^ and CD8^+^ T cells. Analysis of the immune mediators of vaccinated patients demonstrated that levels of IFN-γ, IL-2, IL-12, tumor necrosis factor-α (TNF-α), CCL20, CXCL9, CXCL10 and CXCL14 significantly increased. In contrast, levels of anti-inflammatory and tumor supportive mediators, including IL-6, IL-8, IL-10, TGF-β, CCL2, CCL3, CCL5, vascular endothelial growth factor (VEGF), and matrix metalloproteinases (MMP)-2, MMP-9 decreased in vaccinated patients (72). However, according to available studies, these vaccines may not be effective in patients with advanced cervical cancer (73). This ineffectiveness is probably due to various factors such as immunosuppressive TME and tumor escape mechanisms. Infiltration of myeloid-derived suppressor cells (MDSCs), tumor-associated macrophages (TAMs), Tregs, and expression of PD-1, PDL-1, and CTLA-4 can alter the TME condition in favor of tumor growth and development (42, 48, 51). In this context, combination therapies with chemotherapeutic agents such as carboplatin/paclitaxel or immune checkpoint blockers could improve the effectiveness of E6/E7-based HPV vaccines. These combination therapies can reprogram the TME and regress tumors in patients with advanced cervical cancer (63, 74–76). However, chemotherapy can have several side effects because these compounds affect both tumoral and normal tissues. Furthermore, administering immune checkpoint blockers could be associated with immune-related adverse effects (irAEs), including diarrhea, hypothyroidism, enterocolitis, and pneumonitis (77). On the other hand, the cell distribution difference in the TME known as “hot” and “cold” tumors can affect the effectiveness of combination therapies with immune checkpoint blockers (78, 79).

In the case of peptide-based vaccines, despite the advantages such as ease of production, safety, stability, and cost-effectiveness, one of the main challenges is low immunogenicity, especially in the case of small antigens, which can be solved by adding adjuvants or delivery systems (80, 81). Recently, an investigation reported using a herbal adjuvant termed *Hedyotis diffusa Willd* (Bai Hua She She Cao, BHSSC) extract and rutin as its active compound in peptide-based HPV vaccines enhance vaccine immunogenicity by increasing effector and memory T cell (82). In ISA101, which comprises E6 and E7synthetic peptides, the Montanide ISA 51 adjuvant was used to increase immunogenicity (34). Other delivery systems, such as polystyrene nanoparticles (PSNPs), could also be used to improve CD8^+^ T cell anti-tumor responses in peptide-based HPV vaccines (83). Additionally, epitope selection and the type of adjuvant could be significant in enhancing immune responses following vaccination. For example, using multi-epitope vaccines with appropriate adjuvants can improve the immunogenicity of peptide-based vaccines (1, 84). In this regard, the multi-epitope peptides E6 and E7 proteins are used in ISA101, and the fusion of these oncoproteins partially enhance the vaccine’s function.

## Concluding remarks

Cervical cancer caused by HPV infection is a significant health problem for women worldwide. As a result, vaccination or the emergence of novel therapies can be necessary. Peptide-based vaccines such as ISA101 have appropriate immunogenicity due to their multi-epitope and proper adjuvant. The vaccine contains the two proteins E6 and E7 of HPV 16, which are responsible for a large proportion of cervical cancers, and these peptides are well captured by APCs and presented to CD4^+^ and CD8^+^ T cells. Activation of T cell-dependent responses can eventually lead to tumor regression. However, effective anti-tumor responses following this vaccine do not occur in all individuals, especially in patients with advanced cervical cancer, because factors such as immunosuppressive TME prevent the development of effective anti-tumor immune system responses. In order to overcome this challenge, combining therapies can be a decent approach. For example, chemotherapy and immunotherapy agents in combination with ISA101 increase the effectiveness of cancer therapy.

Interestingly, administrating nivolumab and ISA101 by boosting antigen-specific CD4^+^/CD8^+^ T cells and elevating the efficacy of anti-PD-1 therapy has promising outcomes in human papillomavirus-related head and neck cancers (56). However, the adverse effects of these combination therapies need to be managed. In-depth studies to design more effective vaccines than available HPV vaccines using nanoparticle delivery systems may also increase vaccine therapy efficacy in patients with cervical cancer in the future. However, early diagnosis and initiation of treatment in the early stages of the disease are strongly recommended because as the disease progresses, the effectiveness of monotherapy with vaccines and even combination therapies decreases. In addition to treatment failure, the patient will experience various adverse effects.

## Author contributions

WL and QL, conception, design, and inviting co-authors to participate. HD, JZ and FZ, writing original manuscript draft. YX and YY, review and editing of manuscript critically for important intellectual content and provided comments and feedback for the scientific contents of the manuscript. All authors contributed to the article and approved the submitted version.

## Funding

This work was supported by Public Technology Applied Research Projects of Zhejiang Province (LGF22H060023 to WL), Medical and Health Research Project of Zhejiang Province (2022KY433 to WL, 2020KY998 to QL), Traditional Chinese Medicine Science and Technology Projects of Zhejiang Province (2022ZB382 to WL), Research Fund Projects of The Affiliated Hospital of Zhejiang Chinese Medicine University (2021FSYYZY45 to WL).

## Conflict of interest

The authors declare that the research was conducted in the absence of any commercial or financial relationships that could be construed as a potential conflict of interest.

## Publisher’s note

All claims expressed in this article are solely those of the authors and do not necessarily represent those of their affiliated organizations, or those of the publisher, the editors and the reviewers. Any product that may be evaluated in this article, or claim that may be made by its manufacturer, is not guaranteed or endorsed by the publisher.

## References

[1] Zhang J Fan J Skwarczynski M Stephenson R Toth I Hussein W. Peptide-based nanovaccines in the treatment of cervical cancer: A review of recent advances. Int J Nanomedicine (2022) 17:869. doi: 10.2147/IJN.S269986 35241913 PMC8887913

[2] Deivendran S Marzook KH Radhakrishna Pillai M. The role of inflammation in cervical cancer. Inflamm Cancer (2014) p:377–99. doi: 10.1007/978-3-0348-0837-8_15 24818731

[3] Arbyn M Weiderpass E Bruni L Sanjosé S Saraiya M Ferlay J. Estimates of incidence and mortality of cervical cancer in 2018: a worldwide analysis. Lancet Global Health (2020) 8(2):e191–203. doi: 10.1016/S2214-109X(19)30482-6 PMC7025157 31812369

[4] Bray F Ferlay J Soerjomataram I Siegel R Torre L Jemal A. Global cancer statistics 2018: GLOBOCAN estimates of incidence and mortality worldwide for 36 cancers in 185 countries. CA: Cancer J Clin (2018) 68(6):394–424. doi: 10.3322/caac.21492 30207593

[5] Yagi A Ueda Y Kakuda M Tanaka Y Ikeda S Matsuzaki S. Epidemiologic and clinical analysis of cervical cancer using data from the population-based Osaka cancer RegistryEpidemiologic and clinical analysis of cervical cancer. Cancer Res (2019) 79(6):1252–9. doi: 10.1158/0008-5472.CAN-18-3109 30635276

[6] Bhatla N Aoki D Sharma D Sankaranarayanan R. Cancer of the cervix uteri. Int J gynecol. obstet. (2018) 143:22–36. doi: 10.1002/ijgo.12611 30306584

[7] Pal A Kundu R. Human papillomavirus E6 and E7: the cervical cancer hallmarks and targets for therapy. Front Microbiol (2020) 10:3116. doi: 10.3389/fmicb.2019.03116 32038557 PMC6985034

[8] Yee PC de Souza G,P Khachigian LM. Current and potential treatments for cervical cancer. Curr Cancer Drug Targets (2013) 13(2):205–20. doi: 10.2174/1568009611313020009 23259831

[9] Wright JD Viviano D Powell MA Gibb RK Mutch DG Grigsby PW. Bevacizumab combination therapy in heavily pretreated, recurrent cervical cancer. Gynecol. Oncol (2006) 103(2):489–93. doi: 10.1016/j.ygyno.2006.03.023 16647106

[10] De Sousa LG Rajapakshe K Canales JR Chin RL Feng L Wang Q. ISA101 and nivolumab for HPV-16+ cancer: updated clinical efficacy and immune correlates of response. J Immunother Cancer (2022) 10(2):e004232. doi: 10.1136/jitc-2021-004232 35193933 PMC9066369

[11] van Poelgeest MI Welters MJP Vermeij R Stynenbosch LFM Loof NM Berends-van der Meer DMA. Vaccination against oncoproteins of HPV16 for noninvasive Vulvar/Vaginal lesions: Lesion clearance is related to the strength of the T-cell ResponseVaccine-induced lesion clearance relates to immune response. Clin Cancer Res (2016) 22(10):2342–50. doi: 10.1158/1078-0432.CCR-15-2594 26813357

[12] Melief CJ Welters MJ Vergote I Kroep JR Kenter GG Ottevanger PB. Strong vaccine responses during chemotherapy are associated with prolonged cancer survival. Sci Trans Med (2020) 12(535):eaaz8235. doi: 10.1126/scitranslmed.aaz8235 32188726

[13] Burd EM. Human papillomavirus and cervical cancer. Clin Microbiol Rev (2003) 16(1):1–17. doi: 10.1128/CMR.16.1.1-17.2003 12525422 PMC145302

[14] Torrisi A Del Mistro A Onnis GL Merlin F Bertorelle R Minucci D. Colposcopy, cytology and HPV-DNA testing in HIV-positive and HIV-negative women. Eur J Gynaecol Oncol (2000) 21(2):168–72. 10843478

[15] Walboomers JM Jacobs MV Manos MM Bosch FX Kummer JA Shah KV. Human papillomavirus is a necessary cause of invasive cervical cancer worldwide. J Pathol (1999) 189(1):12–9. doi: 10.1002/(SICI)1096-9896(199909)189:1<12::AID-PATH431>3.0.CO;2-F 10451482

[16] Hillemanns P Wang X. Integration of HPV-16 and HPV-18 DNA in vulvar intraepithelial neoplasia. Gynecol Oncol (2006) 100(2):276–82. doi: 10.1016/j.ygyno.2005.10.003 16300821

[17] Hubert P Herman L Maillard C Caberg JH Nikkels A Pierard G. Defensins induce the recruitment of dendritic cells in cervical human papillomavirus-associated (pre) neoplastic lesions formed in vitro and transplanted in vivo. FASEB J (2007) 21(11):2765–75. doi: 10.1096/fj.06-7646com 17470569

[18] Andersson S Rylanderb E Larssonc B Strandd A Silfversvärde C Wilander E. The role of human papillomavirus in cervical adenocarcinoma carcinogenesis. Eur J Cancer (2001) 37(2):246–50. doi: 10.1016/S0959-8049(00)00376-2 11166153

[19] Huang B Zhu L Wei H Shi H Zhang D Yuan H. Potent neutralizing humanized antibody with topical therapeutic potential against HPV18-related cervical cancer. Front Immunol (2021) 12:2339. doi: 10.3389/fimmu.2021.678318 PMC8264373 34248960

[20] Gagliardi A Porter VL Zong Z Bowlby R Titmuss E Namirembe C. Analysis of Ugandan cervical carcinomas identifies human papillomavirus clade–specific epigenome and transcriptome landscapes. Nat Genet (2020) 52(8):800–10. doi: 10.1038/s41588-020-0673-7 PMC7498180 32747824

[21] Rader JS Tsaih SW Fullin D Murray MW Iden M Zimmermann MT. Genetic variations in human papillomavirus and cervical cancer outcomes. Int J Cancer (2019) 144(9):2206–14. doi: 10.1002/ijc.32038 PMC6450540 30515767

[22] Unger ER Barr E. Human papillomavirus and cervical cancer. Emerg Infect Dis (2004) 10(11):2031. doi: 10.3201/eid1011.04062309 16010736 PMC3329022

[23] Waggoner SE. Cervical cancer. Lancet (2003) 361(9376):2217–25. doi: 10.1016/S0140-6736(03)13778-6 12842378

[24] Cervical cancer treatment (PDQ®)–health professional version (2022). Available at: https://www.cancer.gov/types/cervical/hp/cervical-treatment-pdq#link/_405_toc.

[25] Zepp F. Principles of vaccine design–lessons from nature. Vaccine (2010) 28:C14–24. doi: 10.1016/j.vaccine.2010.07.020 20713252

[26] Eiben GL Da Silva DM Fausch SC Poole CL Nishimura MI Kast WM. Cervical cancer vaccines: recent advances in HPV research. Viral Immunol (2003) 16(2):111–21. doi: 10.1089/088282403322017866 12828864

[27] Toussaint B Chauchet X Wang Y Polack B Gouëllec AL. Live-attenuated bacteria as a cancer vaccine vector. Expert Rev Vaccines (2013) 12(10):1139–54. doi: 10.1586/14760584.2013.836914 24124876

[28] Castle P Maza M. Prophylactic HPV vaccination: past, present, and future. Epidemiol Infection (2016) 144(3):449–68. doi: 10.1017/S0950268815002198 26429676

[29] Hancock G Hellner K Dorrell L. Therapeutic HPV vaccines. Best Pract Res Clin obstet. gynaecol. (2018) 47:59–72. doi: 10.1016/j.bpobgyn.2017.09.008 29108943

[30] HPV vaccination and cervical cancer: A global picture (2022). Available at: https://www.figo.org/news/hpv-vaccination-and-cervical-cancer-global-picture.

[31] Gilboa E. The promise of cancer vaccines. Nat Rev Cancer (2004) 4(5):401–11. doi: 10.1038/nrc1359 15122211

[32] Saxena M Burg SH Melief CJ Bhardwaj N. Therapeutic cancer vaccines. Nat Rev Cancer (2021) 21(6):360–78. doi: 10.1038/s41568-021-00346-0 33907315

[33] Skwarczynski M Toth I. Peptide-based synthetic vaccines. Chem Sci (2016) 7(2):842–54. doi: 10.1039/C5SC03892H PMC5529997 28791117

[34] Van Driel W Ressing ME Kenter GG Brandt RMP Krul EJT van Rossum AB. Vaccination with HPV16 peptides of patients with advanced cervical carcinoma: clinical evaluation of a phase I–II trial. Eur J Cancer (1999) 35(6):946–52. doi: 10.1016/S0959-8049(99)00048-9 10533477

[35] Muderspach L Wilczynski S Roman L Bade L Felix J Small LA. A phase I trial of a human papillomavirus (HPV) peptide vaccine for women with high-grade cervical and vulvar intraepithelial neoplasia who are HPV 16 positive. Clin Cancer Res (2000) 6(9):3406–16. 10999722

[36] Ohno S Okuyama R Aruga A Sugiyama H Yamamoto M. Phase I trial of wilms’ tumor 1 (WT1) peptide vaccine with GM-CSF or CpG in patients with solid malignancy. Anticancer Res (2012) 32(6):2263–9. 22641661

[37] Reuschenbach M Rafiyan M Pauligk C Karbach J Kloor M Sauer ES. Phase I/IIa trial targeting p16INK4a by peptide vaccination in patients with human papillomavirus-associated cancer. Am Soc Clin Oncol (2015) 33:e14030. doi: 10.1200/jco.2015.33.15_suppl.e14030

[38] Coleman HN Greenfield WW Stratton SL Vaughn R Kieber A Moerman-Herzog AM. Human papillomavirus type 16 viral load is decreased following a therapeutic vaccination. Cancer Immunol. Immunother (2016) 65(5):563–73. doi: 10.1007/s00262-016-1821-x PMC4841729 26980480

[39] Rumfield CS Pellom ST Morillon YM Schlom J Jochems C. Immunomodulation to enhance the efficacy of an HPV therapeutic vaccine. J Immunother Cancer (2020) 8(1):e000612. doi: 10.1136/jitc-2020-000612 32554612 PMC7304848

[40] Ma W Melief CJ van der Burg SH. Control of immune escaped human papilloma virus is regained after therapeutic vaccination. Curr Opin Virol (2017) 23:16–22. doi: 10.1016/j.coviro.2017.02.005 28282583

[41] Hasegawa K Ikeda Y Kunugi Y Kurosaki A Imai Y Kohyama S. Phase I study of multiple epitope peptide vaccination in patients with recurrent or persistent cervical cancer. J Immunother (2018) 41(4):201–7. doi: 10.1097/CJI.0000000000000214 29432282

[42] Kenter GG Welters MJP Valentijn ARPM Lo¨wik MJG Berends-van der Meer DMA Vloon PGA. Phase I immunotherapeutic trial with long peptides spanning the E6 and E7 sequences of high-risk human papillomavirus 16 in end-stage cervical cancer patients shows low toxicity and robust immunogenicity. Clin Cancer Res (2008) 14(1):169–77. doi: 10.1158/1078-0432.CCR-07-1881 18172268

[43] Kanodia S Fahey LM Kast WM. Mechanisms used by human papillomaviruses to escape the host immune response. Curr Cancer Drug Targets (2007) 7(1):79–89. doi: 10.2174/156800907780006869 17305480

[44] Wang J Lic Z Gao A Wen Q Sun Y. The prognostic landscape of tumor-infiltrating immune cells in cervical cancer. Biomed Pharmacother (2019) 120:109444. doi: 10.1016/j.biopha.2019.109444 31562978

[45] Heeren AM Punt S Bleeker MCG Gaarenstroom KN van der Velden J Kenter GG. Prognostic effect of different PD-L1 expression patterns in squamous cell carcinoma and adenocarcinoma of the cervix. Modern Pathol (2016) 29(7):753–63. doi: 10.1038/modpathol.2016.64 PMC4931542 27056074

[46] Monk BJ Enomoto T Kast WM McCormack M Tan DSP Wu X. Integration of immunotherapy into treatment of cervical cancer: Recent data and ongoing trials. Cancer Treat Rev (2022) p:102385. doi: 10.1016/j.ctrv.2022.102385 PMC10697630 35413489

[47] O’Malley DM Neffa M Monk BJ Melkadze T Huang M Kryzhanivska A. Dual PD-1 and CTLA-4 checkpoint blockade using balstilimab and zalifrelimab combination as second-line treatment for advanced cervical cancer: an open-label phase II study. J Clin Oncol (2022) 40(7):762–71. doi: 10.1200/JCO.21.02067 PMC8887945 34932394

[48] Kenter GG Welters MJP Valentijn APM Lowik MJG Berends-van der Meer DMA Vloon APG. Vaccination against HPV-16 oncoproteins for vulvar intraepithelial neoplasia. N Engl J Med (2009) 361(19):1838–47. doi: 10.1056/NEJMoa0810097 19890126

[49] de Vos van Steenwijk PJ Ramwadhdoebe TH Löwik MJG van der Minne CE Berends-van der Meer DMA Fathers LM. A placebo-controlled randomized HPV16 synthetic long-peptide vaccination study in women with high-grade cervical squamous intraepithelial lesions. Cancer Immunol Immunother (2012) 61(9):1485–92. doi: 10.1007/s00262-012-1292-7 PMC3427705 22684521

[50] de Vos van Steenwijk PJ van Poelgeest MIE Ramwadhdoebe TH Löwik MJG Berends-van der Meer DMA van der Minne CE. The long-term immune response after HPV16 peptide vaccination in women with low-grade pre-malignant disorders of the uterine cervix: a placebo-controlled phase II study. Cancer Immunol Immunother (2014) 63(2):147–60. doi: 10.1007/s00262-013-1499-2 PMC11028806 24233343

[51] van Poelgeest MI Welters MJP van Esch EMG Stynenbosch LFM Kerpershoek G van Persijn van Meerten EL. HPV16 synthetic long peptide (HPV16-SLP) vaccination therapy of patients with advanced or recurrent HPV16-induced gynecological carcinoma, a phase II trial. J Trans Med (2013) 11(1):1–14. doi: 10.1186/1479-5876-11-88 PMC3623745 23557172

[52] Morrow MP Kraynyak KA Sylvester AJ Shen X Amante D Sakata L. Augmentation of cellular and humoral immune responses to HPV16 and HPV18 E6 and E7 antigens by VGX-3100. Mol Therapy-Oncolytics (2016) 3:16025. doi: 10.1038/mto.2016.25 PMC5147865 28054033

[53] Choi YJ. A Prospective, randomized, multicenter, open-label study of GX-188E, an HPV DNA vaccine, in patients with cervical intraepithelial neoplasia 3A phase II study of a therapeutic HPV DNA vaccine in CIN 3. Clin Cancer Res (2020) 26(7):1616–23. doi: 10.1158/1078-0432.CCR-19-1513 31727676

[54] Kranz LM Diken M Haas M Kreiter S Loquai C Reuter KC. Systemic RNA delivery to dendritic cells exploits antiviral defence for cancer immunotherapy. Nature (2016) 534(7607):396–401. doi: 10.1038/nature18300 27281205

[55] Liu MA. A comparison of plasmid DNA and mRNA as vaccine technologies. Vaccines (2019) 7(2):37. doi: 10.3390/vaccines7020037 PMC6631684 31022829

[56] Massarelli E William W Johnson F Kies M Ferrarotto R Guo M. Combining immune checkpoint blockade and tumor-specific vaccine for patients with incurable human papillomavirus 16–related cancer: a phase 2 clinical trial. JAMA Oncol (2019) 5(1):67–73. doi: 10.1001/jamaoncol.2018.4051 30267032 PMC6439768

[57] Sher Y-P Lee C Liu SY Chen IH Lee MH Chui FF. A therapeutic vaccine targeting HPV E6/E7 with intrinsic toll-like receptor 2 agonist activity induces antitumor immunity. Am J Cancer Res (2018) 8(12):2528–37. PMC6325468 30662809

[58] De Jong A Neill TO Khan AY Kwappenberg KMC Chisholm SE Whittle NR. Enhancement of human papillomavirus (HPV) type 16 E6 and E7-specific T-cell immunity in healthy volunteers through vaccination with TA-CIN, an HPV16 L2E7E6 fusion protein vaccine. Vaccine (2002) 20(29-30):3456–64. doi: 10.1016/S0264-410X(02)00350-X 12297390

[59] Sacco JJ Evans M Harrington KJ Man S Powell N Shan RJ. Systemic listeriosis following vaccination with the attenuated listeria monocytogenes therapeutic vaccine, ADXS11-001. Hum Vaccines Immunotherapeutics (2016) 12(4):1085–6. doi: 10.1080/21645515.2015.1121338 PMC4962931 26618528

[60] Choi CH Choi HJ Lee JW Kang ES Cho D Park BW. Phase I study of a b cell-based and monocyte-based immunotherapeutic vaccine, BVAC-c in human papillomavirus type 16-or 18-positive recurrent cervical cancer. J Clin Med (2020) 9(1):147. doi: 10.3390/jcm9010147 PMC7019768 31948126

[61] Ferrall L Lin KY Roden RBS Hung CF Wu TC. Cervical cancer immunotherapy: Facts and HopesImmunotherapy for cervical cancer. Clin Cancer Res (2021) 27(18):4953–73. doi: 10.1158/1078-0432.CCR-20-2833 PMC8448896 33888488

[62] Glisson B Massarelli E William WN Johnson FM Kies MS Ferrarotto R. Nivolumab and ISA 101 HPV vaccine in incurable HPV-16+ cancer. Ann Oncol (2017) 28:v403–4. doi: 10.1093/annonc/mdx376.002

[63] Gerritsen WR Melief CJ Welters M Vergote I Kroep JR Kenter G. Association of T cell responses after vaccination with HPV16 long peptides for late stage cervical cancer with prolonged survival. Am Soc Clin Oncol (2017) 35:5525–9. doi: 10.1200/JCO.2017.35.15_suppl.5525

[64] Youn JW Hur SY Woo JW Kim YM Lim MC Park SY. Pembrolizumab plus GX-188E therapeutic DNA vaccine in patients with HPV-16-positive or HPV-18-positive advanced cervical cancer: interim results of a single-arm, phase 2 trial. Lancet Oncol (2020) 21(12):1653–60. doi: 10.1016/S1470-2045(20)30486-1 33271094

[65] Burg SHVD van der Sluis TC van Meir H Kroep JR Kenter GG van Poelgeest MIE. Synergistic effects of properly timed HPV16 synthetic long peptide vaccination during standard carboplatin-paclitaxel chemotherapy in animals and in patients with metastatic cervical carcinoma. Cancer Res (2014) 74(19_Supplement):2938–8. doi: 10.1158/1538-7445.AM2014-2938

[66] Influenza type a viruses, centers for disease control and prevention . Available at: http://www.cdc.gov/flu/avianflu/influenza-a-virus-subtypes.htm (Accessed 13 April 2020).

[67] Melief CJM Welters MJ Vergote I Kroep JR Kenter GG Ottevanger NO. Abstract CT002: A strong HPV-specific T-cell response after chemo-immunotherapy for advanced cervical cancer is associated with prolonged survival. Cancer Res (2019) 79(13_Supplement):CT002–2. doi: 10.1158/1538-7445.AM2019-CT002

[68] Roden R Wu TC. How will HPV vaccines affect cervical cancer? Nat Rev Cancer (2006) 6(10):753–63. doi: 10.1038/nrc1973 PMC3181152 16990853

[69] Dochez C Bogers JJ Verhelst R Rees H. HPV vaccines to prevent cervical cancer and genital warts: an update. Vaccine (2014) 32(14):1595–601. doi: 10.1016/j.vaccine.2013.10.081 24606637

[70] Zarchi MK Behtash N Chiti Z Kargar S. Cervical cancer and HPV vaccines in developing countries. Asian Pac J Cancer Prev (2009) 10:969–74. 20192568

[71] Lei J Ploner A Elfström KM Wang J Roth A Fang. HPV vaccination and the risk of invasive cervical cancer. N Engl J Med (2020) 383(14):1340–8. doi: 10.1056/NEJMoa1917338 32997908

[72] Che Y Yang Y Suo J An Y Wang X. Induction of systemic immune responses and reversion of immunosuppression in the tumor microenvironment by a therapeutic vaccine for cervical cancer. Cancer Immunol Immunother (2020) 69(12):2651–64. doi: 10.1007/s00262-020-02651-3 PMC11027467 32607768

[73] Organization WH. WHO position on HPV vaccines. Vaccine (2009) 27(52):7236–7. doi: 10.1016/j.vaccine.2009.05.019 19450645

[74] Domingos-Pereira S Galliverti G Hanahan D Nardelli-Haefliger D. Carboplatin/paclitaxel, E7-vaccination and intravaginal CpG as tri-therapy towards efficient regression of genital HPV16 tumors. J Immunother Cancer (2019) 7(1):1–7. doi: 10.1186/s40425-019-0593-1 31060612 PMC6503370

[75] Peng S Tan M Li Y-D Cheng MA Farmer E Ferrall L. PD-1 blockade synergizes with intratumoral vaccination of a therapeutic HPV protein vaccine and elicits regression of tumor in a preclinical model. Cancer Immunol Immunother (2021) 70(4):1049–62. doi: 10.1007/s00262-020-02754-x PMC7979473 33108473

[76] Peng S Ferrall L Gaillard S Wang C Chi WY Huang CH. Development of DNA vaccine targeting E6 and E7 proteins of human papillomavirus 16 (HPV16) and HPV18 for immunotherapy in combination with recombinant vaccinia boost and PD-1 antibody. MBio (2021) 12(1):e03224–20. doi: 10.1128/mBio.03224-20 PMC7845631 33468698

[77] O’Malley DM Oaknin A Monk BJ Selle F Rojas C Gladieff L. Phase II study of the safety and efficacy of the anti-PD-1 antibody balstilimab in patients with recurrent and/or metastatic cervical cancer. Gynecol Oncol (2021) 163(2):274–80. doi: 10.1016/j.ygyno.2021.08.018 34452745

[78] Gajewski TF Woo SR Zha Y Spaapen R Zheng Y Corrales L. Cancer immunotherapy strategies based on overcoming barriers within the tumor microenvironment. Curr Opin Immunol (2013) 25(2):268–76. doi: 10.1016/j.coi.2013.02.009 23579075

[79] Park JA Cheung N-KV. Limitations and opportunities for immune checkpoint inhibitors in pediatric malignancies. Cancer Treat Rev (2017) 58:22–33. doi: 10.1016/j.ctrv.2017.05.006 28622628 PMC5524462

[80] Whitworth HS Gallagher KE Howard N Mounier-Jack S Mbwanji G Kreimer AR. Efficacy and immunogenicity of a single dose of human papillomavirus vaccine compared to no vaccination or standard three and two-dose vaccination regimens: a systematic review of evidence from clinical trials. Vaccine (2020) 38(6):1302–14. doi: 10.1016/j.vaccine.2019.12.017 31870572

[81] Liu TY Hussein WM Toth I Skwarczynski M. Advances in peptide-based human papillomavirus therapeutic vaccines. Curr topics Medicinal Chem (2012) 12(14):1581–92. doi: 10.2174/156802612802652402 22827526

[82] Song Y-C Huang H-C Chang CY-Y Lee H-J Liu C-T Lo H-Y. A potential herbal adjuvant combined with a peptide-based vaccine acts against HPV-related tumors through enhancing effector and memory T-cell immune responses. Front Immunol (2020) 11:62. doi: 10.3389/fimmu.2020.00062 32153559 PMC7044417

[83] Xiang SD Wilson KL Goubier A Heyerick A Plebanski M. Design of peptide-based nanovaccines targeting leading antigens from gynecological cancers to induce HLA-A2. 1 restricted CD8+ T cell responses. Front Immunol (2018) 9:2968. doi: 10.3389/fimmu.2018.02968 30631324 PMC6315164

[84] de Oliveira LMF Morale MG Chaves AAM Cavalher AM Lopes AS Diniz MDO. Design, immune responses and anti-tumor potential of an HPV16 E6E7 multi-epitope vaccine. PloS One (2015) 10(9):e0138686. doi: 10.1371/journal.pone.0138686 26390407 PMC4577214

